# Successful management of amniotic fluid embolism with cardiac arrest and liver rupture: a case report

**DOI:** 10.3389/fmed.2026.1777512

**Published:** 2026-03-25

**Authors:** Jing Liu, Sirui Ma, Ying Wang, Fu Wei

**Affiliations:** Department of Critical Care Medicine, Xi'an People's Hospital (Xi'an Fourth Hospital), Xi'an, Shaanxi, China

**Keywords:** amniotic fluid embolism, disseminatedintravascular coagulation, ECMO, hemodynamic Instability, spontaneous liver rupture

## Abstract

Amniotic fluid embolism (AFE) is an unpredictable and critical obstetric emergency. Although infrequent, it carries a significant risk of mortality. AFE is typically characterized by abrupt circulatory and respiratory failures, along with disseminated intravascular coagulation (DIC). Another rare complication during pregnancy is spontaneous liver rupture, which poses a serious danger to both the mother and the fetus. AFE generally manifests suddenly during labor, delivery, or shortly after childbirth and is believed to be triggered by the entry of amniotic fluid components into the mother’s bloodstream. Spontaneous liver rupture is more frequently observed in individuals with gestational hypertension, eclampsia, or hemolysis, elevated liver enzymes, and low platelets (HELLP) syndrome. We report the case of a 34-year-old woman (G2P1) with no previous history of hypertension, preeclampsia/eclampsia, or HELLP syndrome. Her delivery was planned for at 40 weeks and 2 days of gestation. During labor, there were sudden drops in fetal heart rate, followed by the emergence of frothy sputum and a loss of consciousness. Although an urgent cesarean section was performed due to a high suspicion of amniotic fluid embolism, the patient still experienced repeated respiratory and cardiac arrests, accompanied by severe postpartum hemorrhage after the surgery. With the aid of venovenous extracorporeal membrane oxygenation (ECMO), surgical exploration revealed a simultaneous liver rupture. Through a collaborative multidisciplinary approach, the patient eventually made a full recovery. The successful management of both AFE and spontaneous liver rupture during pregnancy is exceedingly rare. This report examines pertinent cases and delves into the underlying pathophysiological mechanisms to improve prevention, diagnosis, and treatment strategies for similar occurrences.

## 1 Introduction

Amniotic fluid embolism (AFE) is an uncommon but extremely serious complication during pregnancy, associated with a mortality rate as high as 70–80% (1). The exact cause of AFE is not well-established. The diagnosis is primarily made by ruling out other conditions, as there are currently no widely accepted criteria for its identification. AFE is believed to result from an abnormal immune reaction in the mother triggered when fetal substances enter the lungs (2). Typical symptoms include low oxygen levels, low blood pressure, and issues with blood clotting, which may deteriorate rapidly, potentially leading to cardiac arrest and death (3). Therefore, ensuring hemodynamic stability is vital in treating AFE, with an emphasis on maintaining blood pressure and providing adequate oxygen support (4).

The occurrence of spontaneous liver rupture during pregnancy is remarkably rare, however, it poses significant risks (5). This condition was initially documented by Abercrombie in 1844 (6). Despite improvements in healthcare, mortality rates for both mothers and infants remain alarmingly high. In a study of 67,043 births, spontaneous hepatic rupture was noted in only one instance. However, the incidence increases to 0.05% when accompanied by conditions such as preeclampsia, eclampsia, or HELLP syndrome. However, spontaneous hepatic rupture during pregnancy most commonly involves the right lobe of the liver and is primarily associated with preeclampsia/eclampsia and/or HELLP syndrome, which includes hemolysis, elevated liver enzymes, and low platelet counts (7). Clinically, approximately 30–90% of affected individuals report symptoms such as pain in the right upper quadrant, upper abdominal discomfort, right shoulder pain, nausea, vomiting, abdominal swelling, and signs of hypovolemic shock (6). Owing to its relatively mild clinical signs, AFE can be mistaken for other conditions, significantly complicating the diagnostic process.

We present a case of a 34-year-old pregnant woman from China who was admitted for delivery at 40 weeks and 2 days of gestation, with no significant medical history. Her prenatal assessment indicated that this was her second pregnancy with one previous live birth, and she was experiencing labor in the left occipitoanterior position (LOA). Prenatal evaluation revealed maternal obesity and a small pelvis. Prenatal ultrasound estimated a fetal weight of 4,000 g, and fetal macrosomia was strongly suspected. Given the term gestational age and the patient’s strong desire to deliver vaginally, we planned delivery at this time point.

During the delivery process, she developed an amniotic fluid embolism, resulting in multiple instances of respiratory and cardiac failure, which was further complicated by a spontaneous rupture of the liver. With a collaborative effort among specialists in obstetrics, anesthesiology, general surgery, and intensive care, she was eventually discharged without complications.

## 2 Case report

**Figure 1:**
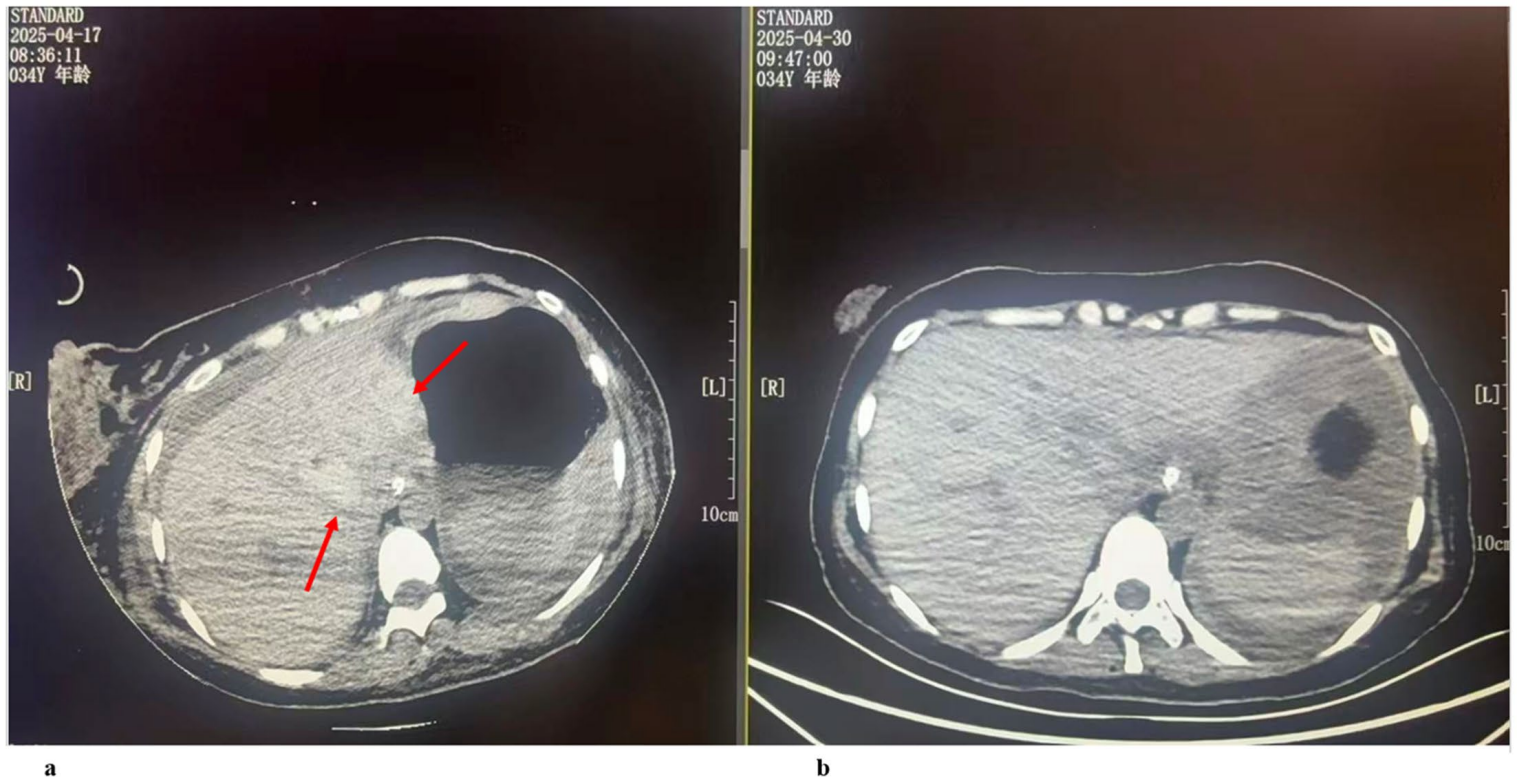
**(a)** On postoperative day 5, abdominal CT of the patient revealed hepatic hemorrhage (indicated by the arrow). **(b)** On postoperative day 18, the patient’s abdominal CT showed that the hemorrhage had been resorbed compared with previous images.

A 34-year-old woman, who had one previous live birth and no other pregnancies, was admitted at 40 weeks and 2 days of gestation due to irregular uterine contractions and mild pain that had lasted for 13 h. Her pregnancy had been normal, with no history of gestational hypertension, diabetes, or other medical issues, and she had no significant family medical history. Prenatal assessments demonstrated normal maternal blood pressure, fetal positioning, and fetal heart rate. An ultrasound examination performed 1 month earlier had revealed polyhydramnios, with the single deepest pocket (SDP) measuring 88 mm; however, a later evaluation confirmed sufficient amniotic fluid volume. Upon admission, her vital signs were temperature of 36.6 °C (97.5 °F), heart rate of 80 bpm, respiratory rate of 20 breaths per minute, and blood pressure of 110/70 mmHg. A thorough physical examination revealed no signs of abdominal injury. Routine laboratory investigations—blood tests, liver and kidney function tests, coagulation parameters, and cardiac ultrasound—showed no significant issues. On the second day after admission, in the absence of any progress in labor, the patient strongly requested intervention. Pitocin was administered at 2.5 units per hour to induce labor. After 20 h of induction, the patient suddenly developed persistent and frequent uterine contractions. Labor signs included 50% cervical effacement, no dilation, fetal head at station S-3, and intact membranes. Following the discontinuation of Pitocin, magnesium sulfate was administered to suppress uterine contractions. Twenty-four hours after the initiation of labor induction, the patient unexpectedly experienced convulsions in all limbs, foaming at the mouth, and loss of consciousness without an obvious cause (heart rate of 60 bpm, blood pressure of 105/67 mmHg, respiratory rate of 26 bpm, oxygen saturation of 85%, and fetal heart rate minimum of 60 bpm). Immediate management included securing the airway, administering oxygen via a mask, continuous monitoring of the fetal heart rate, and continuous intravenous infusion of magnesium sulfate, with a strong suspicion of amniotic fluid embolism. Subsequently, the patient was urgently taken to the operating room for a cesarean section while relevant laboratory tests were conducted. Upon arrival in the operating room, she was in a deep coma, with pupils dilated to approximately 5 mm in diameter, delayed light reflexes, and no spontaneous breathing. Cardiac monitoring showed a heart rate of 30 bpm and no carotid pulse. Tracheal intubation and mechanical ventilation were initiated, along with continuous external cardiac compressions. The emergency protocol for amniotic fluid embolism was activated, involving a multidisciplinary resuscitation team comprising specialists from obstetrics, anesthesiology, neonatology, cardiology, interventional radiology, and critical care medicine. After 6 min of resuscitation, a live female infant weighing 3,550 g was delivered, with Apgar scores of 1, 7, and 8 at 1, 5, and 10 min, respectively. She was transferred to the Neonatal Department for further care. During the surgery, the patient suffered multiple episodes of cardiac and respiratory arrest. Continuous chest compressions were performed, along with an intravenous administration of 400 mg hydrocortisone, repeated doses of 0.5 mg atropine, and 10 mg total epinephrine, together with a 5 mg infusion of milrinone. After 16 min of resuscitation, she regained a spontaneous heart rate of 104 bpm, with oxygen saturation at 77% and blood pressure at 103/68 mmHg (supported by norepinephrine and dopamine infusion). The placenta was delivered spontaneously, but uterine contractions were weak, leading to significant bleeding and clotting failure. Despite attempts at uterine ligation and suturing, bleeding persisted. Coagulation function tests indicated PT: 30.90 s, APTT: 119.70 s, fibrinogen: 0.86 g/L and D-dimer: >20 μg/mL. Biochemical tests showed ALT: 552.34 U/L, AST: 638.70 U/L; a complete blood count revealed WBC: 14.90 × 10^9^/L, Hb: 48 g/L, PLT: 117.00 × 10^9^/L. Echocardiography indicated right ventricular enlargement. The diagnosis considered included amniotic fluid embolism, disseminated intravascular coagulation (DIC), and abnormal liver function, potentially linked to liver damage from the embolism. After discussing with the family, preparations for a hysterectomy was initiated, and a massive transfusion protocol (MTP) was activated. During that time, transfusions included 69 units of packed red blood cells, 7,000 mL of fresh frozen plasma, 70 units of cryoprecipitate, 40 units of platelets, and 22.5 g of fibrinogen, with a total transfusion of approximately 20,800 mL to address coagulation issues. However, the patient continued to experience uncontrollable bleeding, with no improvement in coagulation function. Owing to the patient’s recurrent cardiac arrests, extreme hemodynamic instability, and persistent poor oxygenation, we immediately assembled the ECMO team to prepare to establish veno-arterial extracorporeal membrane oxygenation. Given the patient’s poor coagulation function, the use of anticoagulants during ECMO implementation could potentially exacerbate coagulation disorders and increase bleeding risk. To maintain stable oxygenation and circulation and preserve the patient’s chance of survival, the ECMO team physicians decided to initiate ECMO therapy without anticoagulants. Throughout the procedure, blood counts, coagulation parameters, and activated clotting time (ACT) were closely monitored. ECMO parameters included a blood pump speed of 2,000–2,500 rpm and a blood flow rate of 1.2–2.5 L/min, adjusted in real-time based on clinical conditions. Following successful ECMO initiation, the patient’s blood pressure gradually improved and stabilized; however, bleeding persisted. Hemoglobin levels continued to drop, reaching a low of 45 g/L. A laparotomy was performed by the general surgery team, revealing a rupture in the caudate lobe of the liver, measuring approximately 3.5 cm in length and 1.5 cm in depth, consistent with a Grade III liver injury according to the AAST classification system. Attempts of compression hemostasis using gauze packing were ineffective, following which suturing of the caudate lobe protrusion was performed, which significantly reduced bleeding. Coagulation function recheck results showed PT: 24.70 s, APTT: 121.00 s, fibrinogen: 0.80 g/L, and D-dimer: >20 μg/mL; a complete blood count indicated WBC: 10.70 × 10^9^/L, Hb: 78 g/L, PLT: 117.00 × 10^9^/L (Table 1). The total intraoperative blood loss was approximately 21,000 mL. The patient was safely transferred to the intensive care unit for continued treatment. During hospitalization, she developed multiple organ dysfunction, severe heart failure, severe infection, and psychological trauma. Treatment included antimicrobial therapy, organ function support, hyperbaric oxygen therapy, hypothermic brain protection, blood transfusion to improve coagulation disorders and anemia, stabilization of the internal environment, and psychological counseling. VA-ECMO was successfully discontinued 36 h postoperatively, and the endotracheal tube was removed on the fifth postoperative day. Subsequent abdominal CT revealed heterogeneous density in the left hepatic lobe with multiple slightly hyperdense foci within the liver, suggesting possible hemorrhage (Figure 1a). One week prior to discharge, repeat abdominal CT showed heterogeneous density in the left hepatic lobe with a slightly hyperdense, nodular hemorrhage in the caudate lobe and near the second hepatic hilum of the left lobe, exhibiting decreased density and reduced extent compared to previous imaging (Figure 1b). The patient was ultimately discharged without complications. Total blood products administered during hospitalization included 87 units of packed red blood cells, 14,300 mL of fresh frozen plasma, 82 units of cryoprecipitate, and 60 units of platelets (Table 2). One-month post-discharge, follow-up coagulation studies revealed PT: 12.20 s, APTT: 33.50 s, fibrinogen: 3.34 g/L, and D-dimer: 0.45 μg/mL; a complete blood count indicated Hb: 110.00 g/L and PLT: 298.00 × 10^9^/L, as shown in Table 1. Three months post-discharge, follow-up coagulation tests revealed PT: 11.70 s, APTT: 31.90 s, fibrinogen: 3.89 g/L, and D-dimer: 0.15 μg/mL; a complete blood count indicated Hb: 116.00 g/L and PLT: 312.00 × 10^9^/L, see Table 1. Both results indicated good maternal and infant health with no complications detected.

**Table 1:** Key indicators changes in a parturient with amniotic fluid embolism complicated by hepatic rapture.

Items	Admission	Pre-op	Intra-op	Post-op	POD 1	POD 2	POD 5	POD 8	POD 16	Discharge	1 moth post-disch	3 moth post-disch
Hb (g/L)	109	48	78	82	68	81	94	88	106	101	110	116
PLT (*10^9^/L)	273	101	117	76	45	33	45	118	217	259	298	312
PT (S)	13.90	30.90	24.70	25.40	20.10	16.60	15.20	15.60	16.30	14.40	12.20	11.70
PT (%)	88	23	32	26	44	61	63	68	66	85	89	86
APTT (S)	32.70	119.70	121	142	90	51	29.80	29.90	39.90	38.90	33.50	31.90
TT (S)	16.20	39.50	33.50	24.50	21.80	17.70	15.70	14.60	15.60	16.00	17.20	16.80
FIB (g/L)	38.50	0.86	0.80	1.30	1.58	2.59	3.81	2.86	3.38	4.54	3.34	3.89
D-D (μg/ml)	1.54	>20	>20	>20	>20	12.75	>20	17.75	9.96	9.66	0.45	0.15
ALT (U/L)	7.70	4.59	552.34	291.21	212.90	211.61	171.95	58.10	36.59	32.65	26.56	18.10
AST (U/L)	13.65	13.13	638.70	360.70	259.91	487.80	75.91	27.97	43.47	32.97	27.68	19.95
Alb (g/L)	33.80	29.40	19.47	13.90	20.56	23.24	25.72	28.74	28.90	35.93	40.20	43.35

**Table 2:** Types and amount of transfused blood products during hospitalization

Transfused blood products	Blood transfusion volume
Packed red blood cells (U)	87
PLT (U)	60
Fresh frozen plasma (mL)	14,300
Cryoprecipitated antihemophilic factor (U)	82

## 3 Discussion

Amniotic fluid embolism (AFE) represents a critical complication associated with childbirth and is a primary contributor to maternal fatalities (8). Although the exact pathological process behind AFE is not fully understood, current research suggests that the introduction of amniotic fluid or fetal tissue fragments into the maternal bloodstream acts as a catalyst for this condition (9). Factors that elevate the risk of AFE include advanced maternal age (over 35), multiple pregnancies, preeclampsia or eclampsia, placental issues (such as placenta previa and placental abruption), polyhydramnios, cesarean deliveries, induced labor, cervical tears, uterine or cervical ruptures, artificial rupture of membranes, inappropriate oxytocin administration, uterine hyperstimulation, surgical interventions, premature membrane rupture, fetal complications (such as fetal death or distress), obstructed labor, and abdominal injuries (7). On the day after admission, the patient showed no signs of labor and strongly requested labor induction. The administration of Pitocin, which led to excessive uterine contractions, may have contributed to her AFE. This situation underscores the importance of careful monitoring and management of contractions in women undergoing tocolytic treatment, as contractions induced by medication can be challenging to regulate. Currently, there are no globally recognized diagnostic standards for AFE. Nonetheless, to promote uniformity in AFE reporting, McBride (10) suggested that a diagnosis should fulfill specific criteria: (1) abrupt cardiopulmonary failure or hypotension with respiratory issues, (2) evidence of disseminated intravascular coagulation (DIC), (3) symptoms occurring during labor or within 30 min post-delivery, and (4) absence of fever. Atypical presentations of AFE may arise when some of these criteria are satisfied but the condition cannot be attributed to other causes (10). Many clinical instances of AFE are atypical, with symptoms varying widely. Patients may exhibit early signs such as anxiety, agitation, altered mental status, dyspnea, palpitations, chest discomfort, and even nausea or vomiting (11), necessitating vigilant attention from healthcare providers. AFE typically presents as respiratory and circulatory failure alongside coagulation issues (12), manifesting as sudden breathing difficulties, cardiac arrest, loss of consciousness, and postpartum hemorrhage. When diagnosing AFE, it is essential to exclude other potential causes of similar clinical symptoms, including pulmonary embolism, acute myocardial infarction, cerebrovascular accidents, aortic dissection, anaphylactic shock, placental abruption, and uterine rupture (13). In this case, the patient experienced convulsions, an abrupt loss of consciousness, a significant drop in blood pressure, and cardiopulmonary arrest during the initial stage of labor, followed by DIC—all of which are typical signs of AFE. The patient’s convulsions were initially suspected to be related to eclampsia; however, she exhibited no other signs or symptoms consistent with preeclampsia. Given the timing of cardiac arrest (followed by massive hemorrhage), the clinical diagnosis shifted to amniotic fluid embolism. Differential diagnoses included pulmonary embolism and acute myocardial infarction. The diagnosis of myocardial infarction was excluded, as the patient’s ECG demonstrated no ST‑T changes and troponin was < 0.01 ng/mL.Although the patient’s blood gas analysis indicated hypoxemia complicated with metabolic acidosis and plasma D‑dimer was > 20 μg/mL, lower extremity venous ultrasound showed no evidence of thrombosis, and transthoracic echocardiography revealed no atrial thrombus.Therefore, the diagnosis of pulmonary thromboembolism was not considered in the case. Emergency coagulation tests showed PT 30.90s, APTT 119.70s, and fibrinogen 0.86 g/L (Table 1), indicating acute coagulopathy and further supporting the high likelihood of amniotic fluid embolism. Adhering to the principle of “rescue first, diagnose later”, the prompt initiation of AFE treatment protocols was critical for the patient’s successful resuscitation.

Spontaneous liver rupture is a highly uncommon yet serious complication that can arise during pregnancy, most commonly associated with conditions such as preeclampsia and HELLP syndrome. It may be attributed to increased circulating vasopressin concentrations and heightened vascular sensitivity, which may contribute to vasospasm. Hereditary thrombophilic tendencies may be associated with preeclampsia, eclampsia, and/or HELLP syndrome and other pregnancy complications, though data on this issue remain inconclusive (5). Factors such as elevated blood pressure during pregnancy, multiple gestations, and underlying liver disorders, such as hepatitis, cirrhosis, and hepatic hemangioma, are frequently associated with this phenomenon. The exact pathophysiological processes that lead to spontaneous liver rupture are not fully understood. Research suggests a potential relationship between this condition and maternal age and the number of pregnancies. The condition can manifest after 32 weeks of gestation or in the postpartum period, with a notable increase in cases occurring within the first 15 h after delivery (14). In women experiencing hypertensive disorders during pregnancy, liver rupture may be attributed to vasospasm linked to heightened levels of circulating vasopressin and the body’s increased sensitivity to this hormone. Some researchers propose that frequent uterine contractions during labor, decreased abdominal pressure from fetal movements, and hepatic congestion could all contribute to the onset of spontaneous liver rupture. Additionally, it is important to recognize that the procedures involved in cardiopulmonary resuscitation (CPR) can themselves lead to liver damage. Liver injury is the most prevalent intra-abdominal complication associated with CPR, with reported incidence rates between 0.6 and 3.0% (15). Studies show that liver damage from CPR is more frequently found in the left lobe, while spontaneous liver rupture during pregnancy usually occurs in the right lobe (15, 16). Liver rupture in pregnancy is often associated with the aforementioned risk factors. In cases of uncomplicated pregnancies, spontaneous liver rupture is rare, and its underlying mechanisms remain elusive. We present a case involving a nulliparous primigravida with no prior history of gestational hypertension, eclampsia, HELLP syndrome, or liver disease. Her liver rupture followed multiple instances of CPR, despite the absence of rib or sternal injuries, with the rupture occurring in the caudate lobe, far from the site of CPR compression. Thus, we contend that the risk of liver rupture due to CPR is minimal. The primary factor leading to hepatic rupture in this case was likely circulatory failure and coagulation dysfunction stemming from amniotic fluid embolism. Evidence suggests that disseminated intravascular coagulation (DIC) significantly contributes to spontaneous liver rupture during pregnancy (17). We propose that intense uterine contractions raised intrauterine pressure, allowing amniotic fluid to breach the ruptured uterine sinus wall. This fluid, rich in tissue factor, initiated DIC. As DIC progressed, there was a considerable depletion of platelets and coagulation factors, releasing various inflammatory mediators that caused coagulation dysfunction and bleeding. Microthrombi formed during DIC obstructed hepatic arteriovenous vessels, leading to hepatic congestion. Furthermore, the patient’s abrupt cardiac arrest resulted in circulatory failure, exacerbating hepatic congestion. This chain of events ultimately led to localized liver injury, necrosis with hemorrhage in the hepatic parenchyma, and hepatic steatosis, culminating in liver rupture. It is clear that significant hemodynamic alterations, such as DIC and circulatory failure, can also trigger hepatic rupture during pregnancy. Therefore, it is essential to prioritize hemodynamic stability and swiftly apply appropriate treatments when caring for pregnant patients, especially those in labor, to avert the risk of hepatic rupture.

**Figure 2:**
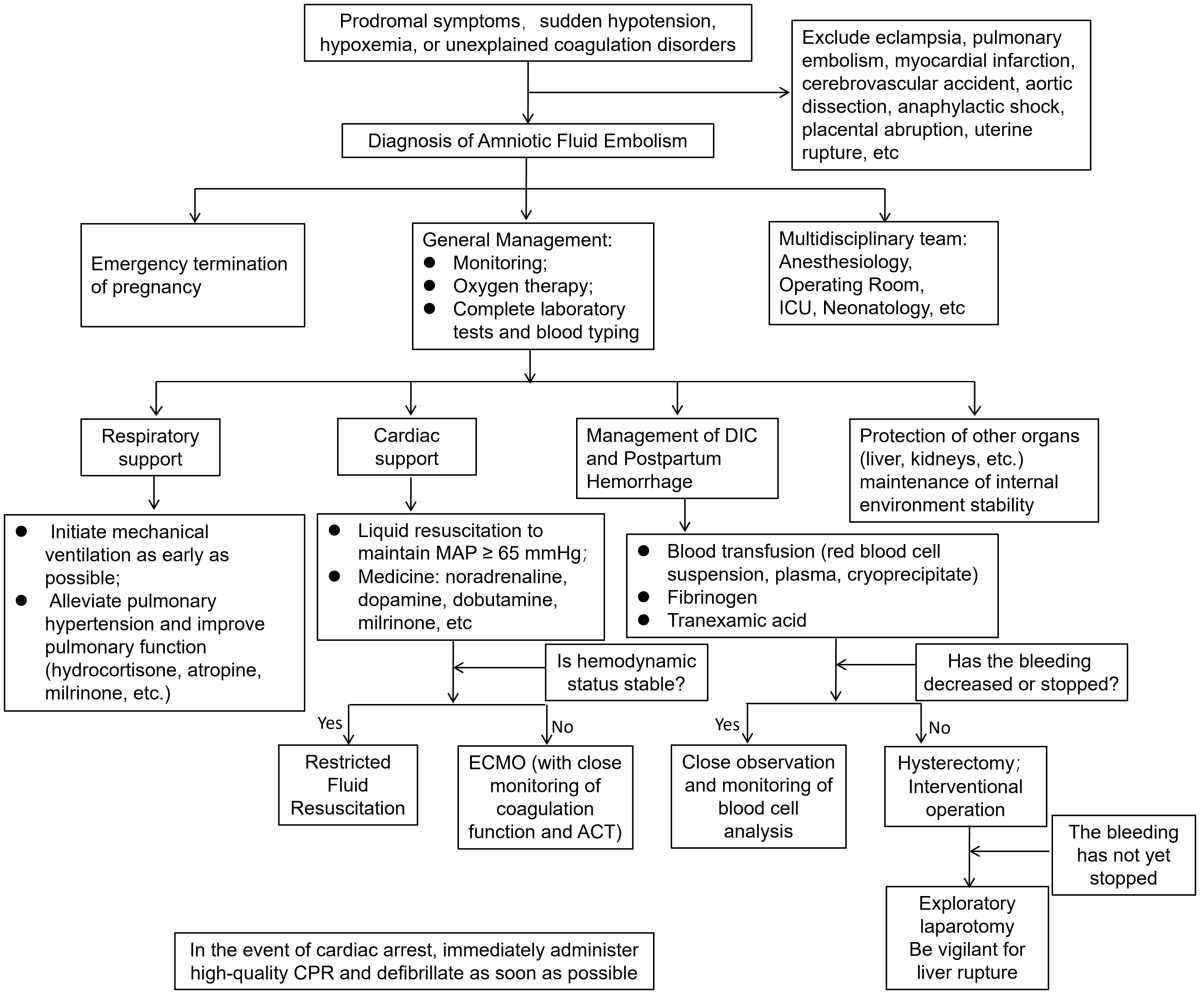
Diagnosis and treatment flowchart for amniotic fluid embolism with cardiac arrest and liver rupture.

Amniotic fluid embolism (AFE) is responsible for 43% of maternal fatalities during pregnancy (18), while liver rupture can lead to maternal and fetal mortality rates as high as 50 to 60% (19, 20). The occurrence of AFE complicated by liver rupture is particularly uncommon. The likelihood of a positive outcome hinges on swift diagnosis and prompt treatment. In this case, we quickly recognized the signs of AFE and activated the treatment protocol (Figure 2). Our approach included a comprehensive care plan with five essential elements: respiratory support, circulatory assistance, management of coagulation issues, obstetric procedures, and thorough monitoring, along with targeted support for organ function. With advancements in technology and the increasing use of VA-ECMO in cardiopulmonary resuscitation, we expect a significant decrease in mortality rates when this technology is applied without delay (21). VA-ECMO serves as a critical life support system for patients experiencing heart and lung failure. ECPR, which utilizes VA-ECMO during resuscitation, is recommended for cardiac arrest cases with reversible causes. Early implementation of ECPR in acute fetal hypoxia situations can substitute for a failing heart, enhancing organ perfusion and optimizing oxygen delivery, making VA-ECMO a valuable adjunctive treatment (10). ECMO is primarily used for patients with refractory cardiogenic shock, with indications including persistently low cardiac output and a cardiac index below 2 L/min/m^2^, despite sufficient intravascular volume and high-dose inotropic support, or hypotension with systolic blood pressure under 90 mmHg (4). However, anticoagulation during ECMO is a frequent clinical challenge, as it is essential to prevent circuit thrombosis and systemic thromboembolism. Nonetheless, anticoagulants in VA-ECMO may worsen bleeding in patients with severe coagulopathy or active hemorrhage. The use of venovenous ECMO in managing AFE remains debated and is not routinely advised (22). Nevertheless, a retrospective study by Aissi James et al. (23) suggests that ECMO can achieve a 70% survival rate in severe cases of AFE during pregnancy. Persistent bleeding, which is the requirement for massive transfusions, and disseminated intravascular coagulation (DIC) should not deter the initiation of ECMO, which should be started without delay. Research by Jaya-Bodestyne (24) indicates that ECPR can enhance oxygenation and improve organ perfusion compared to standard cardiopulmonary resuscitation, potentially reducing complications from prolonged coma. In treating this patient, we promptly initiated VA-ECMO due to ongoing hemodynamic instability that was challenging to manage. We carefully monitored activated clotting time (ACT), platelet counts, coagulation parameters, and other critical indicators. The patient’s hemodynamic condition quickly stabilized, laying a strong foundation for further treatment. Notably, upon entering the operating room after symptom onset, the patient displayed bilateral pupil dilation of 5 mm with delayed light reflexes. An hour later, dilation increased to 6 mm with absent light reflexes. After 26 h of intensive treatment, a physical examination showed signs of pupillary constriction with delayed light reflexes. After 33 h, pupil size improved to 2–3 mm with only slight delays in light reflexes. This improvement may be due to both neuroprotection and the enhanced oxygenation from VA-ECMO. Thus, it is clear that ECMO therapy provides significant clinical advantages for patients experiencing circulatory failure due to AFE, provided that all relevant parameters are meticulously monitored.

This case offers important perspectives on handling amniotic fluid embolism associated with liver rupture; however, it has notable limitations. First, being a single case report restricted its generalizability. Second, the patient exhibited complex and rapidly worsening conditions, which required a sophisticated and challenging treatment approach. Although management was ultimately effective, the strategies used may not be applicable to all situations. Finally, while this study examines possible causes and the underlying mechanisms of amniotic fluid embolism and spontaneous liver rupture, the precise pathophysiology of AFE and liver rupture remains ambiguous. Additional research is needed to clarify these issues.

## 4 Conclusion

In conclusion, we effectively managed an rare case of amniotic fluid embolism that was further complicated by a spontaneous hepatic rupture, providing valuable clinical insights for managing such intricate situations. This case highlights the need for healthcare providers to remain alert for spontaneous liver rupture due to disseminated intravascular coagulation (DIC) in pregnant patients, even when typical risk factors are absent. Further investigation into the underlying pathophysiological mechanisms is essential to improve early detection, develop preventive measures for these challenging conditions, and ultimately reduce morbidity and mortality.

## Data Availability

The raw data supporting the conclusions of this article will be made available by the authors, without undue reservation.
